# Factors associated with health facility utilization during childbirth among 15 to 49-year-old women in Uganda: evidence from the Uganda demographic health survey 2016

**DOI:** 10.1186/s12913-021-07179-5

**Published:** 2021-10-26

**Authors:** Quraish Sserwanja, David Mukunya, Milton W. Musaba, Joseph Kawuki, Freddy Eric Kitutu

**Affiliations:** 1 Programmes Department, GOAL, Khartoum, Sudan; 2 Department of Public Health, Busitema University, Tororo, Uganda; 3 Sanyu Africa Research Institute, Mbale, Uganda; 4 Department of Obstetrics and Gynecology, Busitema University, Tororo, Uganda; 5 Department of Obstetrics and Gynaecology, Mbale Regional Referral and Teaching Hospital, Mbale, Uganda; 6 Centre for Health Behaviours Research, Jockey Club School of Public Health and Primary Care, The Chinese University of Hong Kong, Hong Kong, SAR China; 7 Pharmacy Department, Makerere University School of Health Sciences, Kampala, Uganda; 8 Sustainable Pharmaceutical Systems (SPS) Unit, Makerere University School of Health Sciences, PO Box 7072, Kampala, Uganda

**Keywords:** Health facility, Childbirth, Utilization, Women and Uganda

## Abstract

**Background:**

Almost all maternal deaths and related morbidities occur in low-income countries. Childbirth supervised by a skilled provider in a health facility is a key intervention to prevent maternal and perinatal morbidity and mortality. Our study aimed to establish the factors associated with health facility utilization during childbirth in Uganda.

**Methods:**

We used the Uganda Demographic and Health Survey 2016 data of 10,152 women aged 15 to 49 years. The study focused on their most recent live birth in 5 years preceding the survey. We applied multistage stratified sampling to select study participants and we conducted multivariable logistic regression to establish the factors associated with health facility utilization during childbirth, using SPSS (version 25).

**Results:**

The proportion of women who gave birth at a health facility was 76.6% (7780/10,152: (95% confidence interval, CI, 75.8–77.5). The odds of women aged 15–19 years giving birth at health facilities were twice as those of women aged 40 to 49 years (adjusted odds ratio, AOR = 2.29; 95% CI: 1.71–3.07). Residing in urban areas and attending antenatal care (ANC) were associated with health facility use. The odds of women in the northern region of Uganda using health facilities were three times of those of women in the central region (AOR = 3.13; 95% CI: 2.15–4.56). Women with tertiary education (AOR = 4.96; 95% CI: 2.71–9.11) and those in the richest wealth quintile (AOR = 4.55; 95% CI: 3.27–6.32) had higher odds of using a health facility during child birth as compared to those with no education and those in the poorest wealth quintile, respectively. Muslims, Baganda, women exposed to mass media and having no problem with distance to health facility had higher odds of utilizing health facilities during childbirth as compared to Catholic, non Baganda, women not exposed to mass media and those having challenges with distance to access healthcare.

**Conclusion:**

Health facility utilization during childbirth was high and it was associated with decreasing age, increasing level of education and wealth index, urban residence, Northern region of Uganda, ANC attendance, exposure to mass media, tribe, religion and distance to the nearby health facility. We recommend that interventions to promote health facility childbirths in Uganda target the poor, less educated, and older women especially those residing in rural areas with less exposure to mass media.

## Background

Approximately 830 women die daily from pregnancy related causes globally [1]. More than half of these deaths occur in sub-Saharan Africa [2]. Majority of these deaths are due to direct obstetric complications [2, 3] that can be prevented through early detection and intervention by a skilled healthcare provider [1, 4]. Sadly, a significant proportion of women in low- and middle-income countries still give birth at home unattended to by a skilled health worker [5].

The World Health Organization (WHO) recommends health facility-based childbirth as a key strategy to reduce both maternal and infant mortality, and yet less than half (48%) of childbirths in sub-Saharan Africa occur in a health facility [6]. The remaining half of childbirths occur outside health facilities at home, on the way to the health facility, or at traditional birth attendants’ places that are often unsafe and unhygienic [7]. This low utilization of health facilities during childbirth is likely a reflection of poor affordability and accessibility of health care services [6]. In most low and middle-income countries, home childbirths are conducted by traditional birth attendants (TBAs) because they are cheaper and more readily available for childbirth. However, TBAs are not skilled providers, and are usually under-equipped and lack the appropriate technologies to handle critical birth-related complications, unlike in most health facility childbirths [7].

Over the last two decades, Uganda has achieved a steady reduction in the maternal mortality ratio, currently standing at 336 deaths per 100,000 live births, with substantial variation across regions [8]. However, the current maternal mortality ratio is still high as compared to the Sustainable Development Goals’ target of 70 deaths per 100,000 live births by 2030 [9]. In a low-income country like Uganda, the chances of a safe childbirth are higher when the birth occurs in a health facility than at home or any place outside the health facility [10]. Safe childbirth comprises supportive care, clean delivery practices, timely recognition and management of maternal and neonatal complications [10].

Recent evidence on the determinants of health facility childbirths in Uganda using a nationally representative sample is lacking. Relevant Ugandan studies are either old or have focused on sub-national scope such as districts or regions [11–14]. Mbonye et al. focused on institutional predictors of health facility delivery using data from 2010 national health facility survey [15]. Rutaremwa et al. reported factors associated with a composite outcome - desirable maternal health care package which combined antenatal care (ANC), skilled birth attendance, post-natal care and health facility utilization during childbirth using the 2011 Uganda Demographic and Health Survey (UDHS). Rutaremwa et al. did not assess the predictors of health facility childbirth on its own [16]. Another study by Micah et al. analyzed the 2009–2011 Uganda National Panel Survey data and reported community factors, albeit from a smaller sample size of 3310 [1]. Understanding the determinants of health facility childbirth in Uganda is crucial for prioritizing and stratifying proven interventions that increase utilization of quality maternal health services. Our study aimed to establish the factors associated with utilization of health facilities during childbirth in Uganda.

## Methods

### Data source

We used secondary data of the 2016 nationally representative Uganda Demographic and Health Survey (UDHS) collected from June to December 2016 [8]. The survey was implemented by the Uganda Bureau of Statistics (UBOS) with the technical assistance of Inner City Fund (ICF) International through the USAID-supported MEASURE DHS project [8]. The survey inquired about household members’ and individual characteristics using household questionnaire, women’s questionnaire, men’s questionnaire and biomarker questionnaire [8]. The current study analyzed data that was collected using the women’s questionnaire part of the survey. Women who had given informed consent were asked about the place of delivery for their most recent live birth in the 5 years preceding the survey [8].

### Study setting

Uganda has a tiered health system, from the highest level of national tertiary referral hospitals to the lowest at the community [17, 18]. It is a mixed health system where public and private health providers co-exist [18]. Over 25 years ago, Uganda adopted a decentralized approach to service delivery with local government at districts overseeing, managing and mobilizing resources for service delivery, including healthcare services [18]. The Ugandan government abolished user fees in 2001 in all public health facilities. However, the health service delivery and utilization still face multiple challenges including inadequate staffing, low pay, shortage of medicines and poor infrastructure [18].

### Study sampling and participants

UDHS employed two-stage cluster sampling technique where the census enumeration areas were the primary sampling units while households were the second stage of sampling [8]. The enumeration areas were selected from the 2014 population and housing census sample frame [8]. Women aged 15 to 49 years who were either permanent residents or spent the night preceding the survey in the selected household were eligible for inclusion in the Uganda’s demographic health survey 2016 [8]. Of the 18,506 women who consented and filled in the questionnaires, 10,152 responded to the question about place of child birth considering their most recent live birth in the 5 years preceding the survey [8].

### Variables

#### Dependent variables

Health facility delivery was defined as birth that occurred inside a health facility, whether private or government. Childbirth outside a health facility was defined as birth that occurred outside a health facility including at the home of the woman’s, relatives’, or traditional birth attendants’ on the way to the health facility. Birth outside health facility was coded as zero (0) while health facility delivery was coded as one (1).

#### Independent variables

**Fig. 1:**
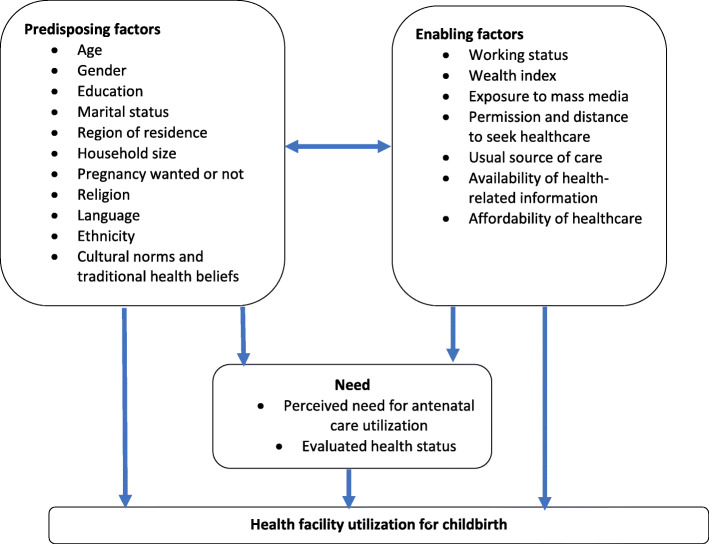
Conceptual framework for the factors associated with health facility utilization for childbirth

In this study, we conceptualized the factors associated with decision making regarding the place of childbirth by the mother using a conceptual framework heavily influenced by Andersen’s behavioral model of health service use. Borrowing from this theoretical framework, we developed a conceptual framework (See Fig. 1). Additionally, only variables that are collected in the routine DHS were examined in our study. According to our conceptual framework, utilization of health facility during child birth could be a function of three categories of factors, namely: predisposing factors (socio-demographic factors), enabling factors (e.g., wealth index) and healthcare needs [19–21]. The predisposing factors in the conceptual framework are: age, level of education, region of residence, place of residence, religion, marital status, household size, sex of household head and tribe. Wealth index, working status, exposure to mass media, problems seeking permission and distance to the nearest health facility as an indicator of access were considered as enabling factors, while visiting the health facility for antenatal care was included in the model as a proxy for the perceived need, as illustrated in Fig. 1.

Maternal age was categorized as; (15–19 years, 20–29 years, 30–39 years, 40–49 years) [22]. Wealth index is a measure of relative household economic status and was calculated by UDHS from information on household asset ownership using Principal Component Analysis, which was further categorized into poorest, poorer, middle, richer and richest quintiles [8]. Place of Residence was categorized into urban and rural.

Region was categorized into four; Northern (Teso, Karamoja, Lango, Acholi, West Nile), Central (Kampala, Central 1 and Central 2), Eastern (Busoga, Bugishu and Bukedi) and Western (Tooro, Ankole, Bunyoro and Kigezi) [23]. Level of Education was categorized into no education, primary education, secondary and tertiary education. Household Size was categorized as less than six members and six and above members (based on the national average and the dataset average of six members per household). Sex of household head was categorized as male or female, working status categorized as: not working and working while marital status as married (this included those in formal and informal unions) and not married. ANC attendance was categorized as yes (for any woman who attended ANC regardless of the frequency) and no (for those who did not attend ANC at all). Religion was categorized as Muslims, Anglican, Catholics, Pentecostal, and others while tribe was categorized as Acholi, Baganda, Bagisu, Bakiga, Banyankole, Basoga, Langi, Itesot and others. Problems with access to care (either seeking permission or distance to health facility) were categorized as big problem and no big problem while exposure to any of the three-mass media avenues (TV, radio, and newspapers) was categorized as yes and no and whether pregnancy was wanted was categorized as no, later and then.

### Statistical analysis

In order to account for the multi-stage cluster study design, we used complex sample package of SPSS (version 25.0) statistical software and adjusted the data using sampling weights, primary sampling units, and strata. We carried out the weighted count to account for the unequal probability sampling in different strata and to ensure representativeness of the survey results at the national and regional level. We performed Chi-square tests to determine the association between the independent variables and the outcome variable (place of childbirth). Logistic regression analyses were conducted to determine the strength of associations between independent variables and the outcome variable, after adjusting for extraneous variables.

Before multivariable logistic regression, each exposure/predictor (independent variable) was assessed separately for its association with the outcome variable using bivariate logistic regression by reviewing the crude odds ratio (COR), 95% confidence interval (CI) and *p*-values. The conceptual framework was relied upon to select variables for the multivariable logistic regression. Additionally, independent variables with a *p-*value ≤0.20 at the bivariate analysis [24] were included in the final multivariable logistic regression model to assess the independent effect of each variable on the outcome variable. All variables in the model were assessed for collinearity. The highest variance inflation factor (VIF) observed was 1.96. Adjusted odds ratios (AOR), 95% confidence intervals (CI) and *p-*values were calculated with statistical significance level set at *p-*value < 0.05. Sensitivity analysis was conducted with the multivariable model by examining each mass medium and parameter of access to healthcare for their individual effects on place of childbirth.

## Results

### Study sample characteristics

A total of 10,152 women were included in the analysis **(**Table 1). Of these, 7780 (76.6%) (95% confidence interval, CI: 75.8–77.5) had their most recent deliveries at a health facility (public or private health facility) and 2372 (23.4%) (95% CI: 22.5–24.2) outside a health facility. Majority of the women were residing in rural areas (76.9%), in male headed households (73.1%), were married (81.3%), and had work (83%). Additionally, they were less than 30 years of age (59.5%), had primary as highest level of education (60%) and had attended ANC (98.1%). Majority of the women were Catholic (39.4%) and had problems with accessing health care due to distance to the nearby health facility (39.8%) and 15.4% were Baganda by tribe. Majority of the women (77.6%) had exposure to one of the three mass media (radio, TV and newspapers). Separately, radio was the most exposed to mass media at 73.7% with only 26.3% not exposed to radio compared to 80.6 and 71% not being exposed to newspapers and TV respectively. The mean age and household size were 28.5 + 7.14 and 6.0+ 2.80 respectively. The prevalence of skilled birth attendance (SBA) among women who had childbirth at home was 8.9% (95% CI: 7.9–10.2) compared to 96.7% (95% CI: 96.4–97.2) for those that had childbirth at a health facility. Results of the chi-square test also showing the distribution of health facility utilization by background characteristics are shown in Table 2.

**Table 1:** Socio-demographic characteristics of women in Ugandan as per the 2016 UDHS

Characteristics	***N*** = 10,152	%
Age		
15 to 19	823	8.1
20 to 29	5217	51.4
30 to 39	3214	31.7
40 to 49	899	8.9
**Residence**		
Urban	2346	23.1
Rural	7806	76.9
**Region**		
Western	2559	25.2
Eastern	2113	20.8
Central	2805	27.7
Northern	2675	26.3
**Sex household head**		
Female	2726	26.9
Male	7426	73.1
**Household Size**		
6 and above	5062	49.9
Less than 6	5090	50.1
**Working status**^**a**^		
Not working	1720	16.9
Working	8425	83.1
**Marital status**		
Married	8256	81.3
Not married	1896	18.7
**Education Level**		
No Education	1061	10.5
Primary Education	6091	60.0
Secondary Education	2285	22.5
Tertiary	715	7.0
**Wealth Index**		
Poorest	2117	20.9
Poorer	2074	20.4
Middle	1921	18.9
Richer	1862	18.3
Richest	2178	21.5
**Place of Delivery**		
Health Facility	7780	76.6
Home	2372	23.4
**ANC Attendance**		
Yes	9957	98.1
No	195	1.9
**Religion**		
Catholics	4003	39.4
Anglican	3154	31.1
Muslims	1409	13.9
Pentecostal	1285	12.7
Others	301	3.0
**Tribes**		
Baganda	1564	15.4
Itesot	781	7.7
Langi	575	5.7
Basoga	788	7.8
Banyankole	1069	10.5
Bakiga	691	6.8
Bagisu	508	5.0
Others	3679	36.2
Acholi	497	4.9
**Exposure to newspapers**		
No	8188	80.6
Yes	1964	19.4
**Exposure to TV**		
No	7211	71.0
Yes	2941	29.0
**Exposure to Radio**		
No	2667	26.3
Yes	7485	73.7
**Exposure to any of the mass media**		
No	2278	22.4
Yes	7874	77.6
**Permission to access healthcare**		
Big problem	528	5.2
Not big problem	9624	94.8
**Distance to health facility**		
Big problem	4039	39.8
Not big problem	6113	60.2
**Access to healthcare**		
Big problem	364	3.6
Not big problem	9789	96.4
**Pregnancy wanted**		
No	1131	11.1
Later	3417	33.7
Then	5604	55.2

^a^ working status missing 7 respondents

**Table 2:** Distribution of health facility utilization by background characteristics among women in Uganda

Characteristics	Home ***N*** = 2372 n (%)	Health facility ***N*** = 7780 n (%)	***P***-Value
**Household Head**			0.082
Male	1768 (74.5)	5659 (72.7)	
Female	604 (25.5)	2121 (27.3)	
**Wealth Index**			< 0.001*
Poorest	682 (28.8)	1435 (18.4)	
Poorer	680 (28.7)	1394 (17.9)	
Middle	510 (21.5)	1411 (18.1)	
Richer	356 (15.0)	1506 (19.4)	
Richest	144 (06.1)	2035 (26.2)	
**Working Status**^**a**^			< 0.001*
Working	2033 (85.7)	6392 (82.2)	
Not Working	338 (14.3)	1382 (17.8)	
**Education Level**			< 0.001*
No Education	359 (15.1)	702 (09.0)	
Primary	1738 (73.3)	4353 (56.0)	
Secondary	251 (10.6)	2034 (26.1)	
Higher	24 (01.0)	691 (8.9)	
**Region**			< 0.001*
estern	731 (30.8)	1828 (23.5)	
Eastern	589 (24.8)	1524 (19.6)	
Central	494 (20.8)	2311 (29.7)	
Northern	558 (23.5)	2117 (27.2)	
**Marital Status**			0.130
Married	1954 (82.4)	6301 (81.0)	
Not Married	418 (17.6)	1479 (19.0)	
**Age**			< 0.001*
15 to 19	151 (06.4)	671 (08.6)	
20 to 29	1072 (45.2)	4145 (53.3)	
30 to 39	839 (35.4)	2375 (30.5)	
40 to 49	309 (13.0)	590 (7.6)	
**Residence**			< 0.001*
Rural	2125 (89.6)	5682 (73.0)	
Urban	247 (10.4)	2098 (27.0)	
**Household Size**			< 0.001*
Six and above	1321 (55.7)	3740 (48.1)	
Less than 6	1051 (44.3)	4040 (51.9)	
**ANC**			< 0.001*
No	111 (4.7)	84 (1.1)	
Yes	2261 (95.3)	7996 (98.9)	
**Religion**			< 0.001*
Catholics	975 (41.1)	3028 (38.9)	
Anglican	779 (32.8)	2375 (30.5)	
Muslims	226 (9.5)	1183 (15.2)	
Pentecostal	315 (13.3)	970 (12.5)	
Others	77 (3.2)	224 (2.9)	
**Tribes**			< 0.001*
Baganda	190 (8.0)	1375 (17.7)	
Itesot	191 (8.1)	590 (7.6)	
Langi	166 (7.0)	410 (5.3)	
Basoga	124 (5.2)	664 (8.5)	
Banyankole	252 (10.6)	817 (10.5)	
Bakiga	265 (11.2)	426 (5.5)	
Bagisu	178 (7.5)	330 (4.2)	
Others	947 (39.9)	2731 (35.1)	
Acholi	59 (2.5)	437 (5.6)	
**Exposure to newspapers**			< 0.001*
No	2171 (91.5)	6016 (77.3)	
Yes	201 (8.5)	1764 (22.7)	
**Exposure to Radio**			< 0.001*
No	812 (34.2)	1855 (23.8)	
Yes	1560 (65.8)	5925 (76.2)	
**Exposure to TV**			< 0.001*
No	2016 (85.0)	5194 (66.8)	
Yes	356 (15.0)	2586 (33.2)	
**Exposure to mass media**			< 0.001*
No	750 (31.6)	1528 (19.6)	
Yes	1622 (68.4)	6252 (80.4)	
**Permission to access healthcare**			0.001*
Big problem	154 (6.5)	374 (4.8)	
Not big problem	2218 (93.5)	7407 (95.2)	
**Distance to health facility**			< 0.001*
Big problem	1221 (51.5)	2817 (36.2)	
Not big problem	1151 (48.5)	4963 (63.8)	
**Access to healthcare**			< 0.001*
Big problem	113 (4.8)	251 (3.2)	
Not big problem	2259 (95.2)	7529 (96.8)	
**Pregnancy wanted**			< 0.001*
No	368 (15.5)	762 (9.8)	
Later	784 (33.1)	2633 (33.8)	
Then	1220 (51.4)	4385 (56.4)	

^a^Working status had 7 missing values.

* Significant at *p*-value < 0.05

### Factors associated with health facility delivery

After adjusting for other extraneous variables, factors associated with health facility delivery were: age, level of education, region of residence, wealth index, ANC attendance, religion, tribe, exposure to mass media and problems with distance to health facility as indicated in Table 3. Women in younger age groups and those with higher levels of education had higher odds of utilizing a health facility during childbirth. The odds of women with tertiary and secondary education attainment utilizing a health facility for childbirth were almost five-fold (4.96) and two-fold as those among women with no education attainment, respectively. The effect of region of residence remained significant in the multivariable model, with the odds of utilizing a health facility for childbirth for women living in the northern region being thrice (3.13) those of women from the central region. The odds of health facility childbirth among women from the richest households and urban areas were almost five-fold (4.55) and almost two-fold (1.45) those among women in the poorest quintile and rural areas, respectively. ANC attendance, a factor depicting perceived need remained significant in multivariable analysis; the odds of health facility utilization during childbirth among women who attended ANC were almost four-fold (3.56) those of women who had not attended ANC. Religion and tribe were also significantly associated with place of childbirth. Muslims had higher odds of utilizing health facilities compared to those of Catholics. Being Itesot, Langi, Munyankole, Mukiga and Mugisu had less odds of utilizing a health facility for childbirth as compared to Baganda, as detailed in Table 3**.** Exposure to mass media was significantly associated with health facility utilization with women exposed to mass media having higher odds (1.24) as compared to those women who were not exposed to mass media.

**Table 3:** Factors associated with health facility utilization at birth among women in Uganda

Characteristics	Crude odds ratio, COR (95%CI), (***N*** = 10,152)	***p***-value	Adjusted odds ratio, AOR (95%CI), (***N*** = 10,152)	***p***-value
**Age**		< 0.001*		< 0.001*
40 to 49	1		1	
30 to 39	**1.48 (1.24–1.78)**		**1.29 (1.06–1.58)**	
20 to 29	**2.03 (1.71–2.41)**		**1.56 (1.26–1.93)**	
15 to 19	**2.32 (1.80–3.00)**		**2.29 (1.71–3.07)**	
**Education Level**		< 0.001*		< 0.001*
No Education	1		1	
Primary	**1.28 (1.08–1.52)**		1.07 (0.89–1.28)	
Secondary	**4.14 (3.27–5.25)**		**2.09 (1.62–2.70)**	
Tertiary	**14.70 (8.21–26.32)**		**4.96 (2.71–9.11)**	
**Marital Status**		0.130		0.937
Not married	1		1	
Married	0.91 (0.80–1.04)		1.01 (0.85–1.19)	
**Working Status**		< 0.001*		0.174
Working	**1**		1	
Not working	**1.30 (1.06–1.59)**		1.15 (0.94–1.39)	
**Region**		< 0.001*		< 0.001*
Central	1		1	
Western	**0.53 (0.40–0.71)**		**1.46 (1.09–1.95)**	
Eastern	**0.55 (0.42–0.72)**		1.35 (0.97–1.88)	
Northern	**0.81 (0.62–1.06)**		**3.13 (2.15–4.56)**	
**Household Size**		< 0.001*		0.364
Less than 6	**1**		1	
Six and above	**0.74 (0.65–0.83)**		0.94 (0.81–1.08)	
**Wealth Index**		< 0.001*		< 0.001*
Poorest	**1**		1	
Poorer	0.98 (0.82–1.16)		**1.25 (1.04–1.51)**	
Middle	**1.32 (1.07–1.61)**		**1.74 (1.40–2.17)**	
Richer	**2.01 (1.61–2.51)**		**2.24 (1.75–2.87)**	
Richest	**6.72 (5.09–8.88)**		**4.55 (3.27–6.32)**	
**Residence**		< 0.001*		0.002*
Rural	**1**		**1**	
Urban	**3.17 (2.52–3.99)**		**1.45 (1.15–1.83)**	
**Sex of household head**		0.082		0.831
Male	1		1	
Female	1.10 (0.97–1.24)		1.02 (0.87–1.18)	
**ANC**		< 0.001*		< 0.001*
No	**1**		**1**	
Yes	**4.47 (3.04–6.58)**		**3.56 (2.43–5.23)**	
**Religion**		< 0.001*		0.329
Catholics	**1**		**1**	
Anglican	0.98 (0.85–1.14)		1.07 (0.93–1.24)	
Muslims	**1.68 (1.33–2.13)**		**1.26 (1.02–1.56)**	
Pentecostal	0.99 (0.80–1.24)		1.06 (0.86–1.31)	
Others	0.94 (0.65–1.36)		1.08 (0.73–1.62)	
**Tribes**		< 0.001*		< 0.001*
Baganda	**1**		**1**	
Itesot	**0.43 (0.29–0.62)**		**0.54 (0.35–0.84)**	
Langi	**0.34 (0.24–0.49)**		**0.39 (0.25–0.61)**	
Basoga	0.74 (0.50–1.09)		1.11 (0.69–1.80)	
Banyankole	**0.45 (0.33–0.62)**		**0.63 (0.45–0.88)**	
Bakiga	**0.22 (0.16–0.30)**		**0.38 (0.26–0.55)**	
Bagisu	**0.26 (0.17–0.38)**		**0.39 (0.24–0.62)**	
Others	**0.40 (0.30–0.53)**		**0.62 (0.46–0.83)**	
Acholi	1.02 (0.67–1.54)		1.27 (0.76–2.10)	
**Exposure to mass media**		< 0.001*		0.002*
No	**1**		1	
Yes	**1.89 (1.66–2.16)**		**1.24 (1.08–1.42)**	
**Access to healthcare**				0.107
Big problem	**1**	< 0.001*	**1**	
Not big problem	**1.51 (1.17–1.93)**		1.26 (0.95–1.66)	
**Pregnancy wanted**		< 0.001*		0.464
No	**1**		1	
Later	**1.62 (1.37–1.91)**		1.03 (0.84–1.25)	
Then	**1.73 (1.46–2.06)**		1.11 (0.92–1.35)	

bold* Significant at *p*-value < 0.05, *AOR* Adjusted odds ratio, *COR* Crude Odds Ratio

Most of the study participants were from households headed by men and there was no difference in proportions of households headed by men and marital status by place of childbirth. There was a difference in the distribution of households by wealth index, working status, education level, region of residence, urban/rural distribution, age, and household size between health facility child birth and home childbirth. Also, there was statistical difference by place of birth for ANC, tribe, exposure to newspapers, radio, mass media, permission to access healthcare distance to health facility and whether the pregnancy was wanted or not (see Table 2).

### Sensitivity analysis

When each of mass medium (radio, TV and newspapers) and parameters of problems accessing healthcare, respectively, were added in the multivariable model individually, age, level of education, region, wealth index, residence, ANC attendance, religion and tribe remained significantly associated with health facility childbirth. Regarding exposure to mass media, only exposure to newspapers (AOR = 1.34; 95% CI: 1.09–1.65) and radio (AOR = 1.18; 95% CI: 1.04–1.34) were associated with place of childbirth. Unlike the primary analysis where problems accessing healthcare were not significantly associated with place of childbirth, in the sensitivity analysis, the odds (AOR = 1.42; 95% CI: 1.25–1.60) of utilizing a health facility during childbirth among women who had no big problem with distance to access a health facility were 1.42 times those of women who had a big problem with distance.

## Discussion

This study examined the factors associated with health facility delivery among Ugandan women. We found that almost 80% of the women gave birth in a health facility. This proportion is higher than national studies that have used similar DHS survey data in Kenya [5], Indonesia [25], Ghana [26] and in other regional studies done in South Sudan, Kenya and Ethiopia [2, 27, 28]. Studies that showed a lower proportion of health facility utilization during childbirth compared to our study were done earlier than our study (2011–2014) except Tongun et al. in South Sudan [2]. The earlier studies reported lower proportions likely because health facility utilization during childbirth has been shown to increase with time. Furthermore, the differences in the health facilities’ access, health system capacity and economic development among these countries could also explain the observed differences. The significantly low proportion of health facility utilization during childbirth (25%, lowest in the region) shown by Tongun et al. in South Sudan could be attributed to the fact that the study was done when the country was experiencing insecurity due to the civil unrest, which negatively affected health facility access and led to destruction of infrastructure [2]. South Sudan received independence from Sudan nine (9) years ago and it is still grappling with a weak health infrastructure and system.

Age, level of education, region, wealth index, residence, ANC attendance, religion, tribe, exposure to mass media and problems with distance to health facility were significantly associated with health facility delivery utilization. Younger women had higher odds of utilizing a health facility for childbirth compared to their older counterparts. Older women tend to have more traditional cohorts hence can easily resist modern health care services [29, 30], and some tend to have a sense of having gained enough experience when it comes to childbirth, hence have less fear for negative pregnancy outcomes associated with delivery outside a health facility [31, 32]. Age as a predictor of health facility utilization during childbirth has also been evidenced by other studies [5, 29, 32–34]. Women with secondary and post-secondary education had higher odds of giving birth from a health facility compared to women with no education. Women with higher levels of education have been shown to be more receptive to new health related information, have better maternal health literacy and increased awareness of available health resources. They also seem to have better decision-making abilities, more financial resources and access to health insurance. Taken together, these factors have been shown to increase health facility-based deliveries [2, 27, 29, 35]. With improved maternal health literacy, women become more informed about maternal health care issues which enables them to make positive health care decisions [26]. Maternal education as a predictor of health facility utilization during childbirth has also been evidenced by other studies [2, 26–28, 36]. Therefore, the government of Uganda needs to intensify girl-child education to at least secondary level and also improve or start maternal health programs targeting the less educated women.

The odds of women from the northern region utilizing health facilities during childbirth were three times those in the central region. This is a surprising finding because the central region is more advanced with a high concentration of health facilities and health care workers. However, our finding is similar to that of Rutaremwa et al. who analyzed the utilization of maternal health services with the 2011 UDHS data [16]. Rutaremwa et al. using Kampala (the capital) as the reference showed that even if the other four regions were less likely to utilize maternal health services compared to Kampala, northern region had the highest likelihood of utilizing the desirable maternal health services package compared to the central, western and eastern regions [16]. The differences in health facilities’ accessibility, sociocultural context and economic development could have contributed to the observed regional differences in utilization of health facility at birth [16]. Following the civil war, the northern region has had many interventions and humanitarian aid mainly targeting maternal health services improvement [37]. The other possible explanation could be that many people were residing in internally displaced peoples’ camps and these usually have health facilities provided freely near the camps [37]. However, further research is needed to explore the increased utilization of health facilities at birth in northern Uganda. Region as a predictor of health facility delivery has been reported in studies done in similar contexts [5, 16, 32].

Women belonging to higher wealth quintiles had higher odds of giving birth from a health facility compared to those in the poorest wealth quintile. Given that Uganda has free health care services [18], our results suggest that, apart from the cost of health services, other economic factors influence the choice of place of delivery. This is consistent with findings from other studies, which have reported economic factors such as transportation costs, and miscellaneous fees paid for healthcare to influence the women’s decision regarding the place of childbirth [26, 38]. Hence, there is a need for gaining a deeper understanding of how financial status influences women’s decision regarding choice of place of childbirth. Women belonging to the lower wealth quintiles are more likely to have difficulties in meeting transport and indirect costs related to childbirth in government facilities which prevents them from utilizing health facilities for childbirth [2], and this is further worsened by the high costs of private health facilities [9]. Wealth index has also been shown to be a predictor of health facility deliveries in previous studies done in Kenya, Ghana and South Sudan [2, 5, 26, 27].

Urban women had higher odds of giving birth from a health facility compared to rural women. Urban areas usually have more and better health facilities than rural areas hence better access to healthcare [25, 26, 39]. This proximity to health facilities in urban areas ensures better quality of maternal health services through quick referrals and easier use of multidisciplinary teams [26]. In addition, women in urban areas are usually more financially stable, and have more access to media promoting good maternal health [26, 39]. Place of residence has been shown in other studies done in Indonesia, Ethiopia, Nigeria, Kenya and Ghana to influence choice of place of childbirth [5, 16, 25, 26, 39, 40]. With evidence of the association between residence and place of childbirth presented in the current, the government efforts should prioritize improving rural health services in Uganda.

Women who had attended antenatal care had higher odds of utilizing health facilities for childbirth compared to those who had not attended antenatal care. Visiting of health facilities for antenatal care ensures that women get health education sessions regarding the benefits of institutional delivery and creates rapport between the health workers and the pregnant women [27, 41]. The health education and counselling sessions during antenatal care visits also ensures that women make birth preparedness and complication readiness plans which contributes to increased health facility utilization during childbirth [36]. Antenatal care attendance has also been shown to be a predictor of health facility utilization during childbirth in studies done in similar contexts [11, 27, 28, 36].

Women that belonged to other ethnic groups (Itesot, Langi, Banyankole, Bakiga, Bagisu and others) were had lower odds of utilizing health facilities for childbirth compared to Baganda. In addition, contrary to studies done in Nigeria, Ghana, Guinea and Bangladesh [42–45] that showed Muslim women to have lower odds of utilizing health facilities for childbirth compared to Christian women, our study showed Muslims to have higher odds of using health facilities for childbirth. Religious and ethnic cultural values and norms have been shown to influence choice of the place of childbirth [16, 42, 44–48]. Plausible explanations include differences in levels of literacy, empowerment, and autonomy among the different ethnic groups and differences in religious teachings and related preferences, and choices regarding the use of modern versus traditional medicine [44, 45]. Rutaremwa et al. showed that some cultural norms make women to adhere to very traditional childbirth practices and believe that pregnancy is a test of endurance [16]. Thus, in some Ugandan tribes, women are considered to be strong and independent if they can handle the childbirth process by themselves which discourages women from seeking professional maternity care and opt for childbirth outside health facilities [16, 44, 46]. However, there is need for further exploration of the impact of ethnicity and religion on the place of childbirth in Uganda. Maternal health policy makers should consider systematic and deliberate involvement of tribal and religious leaders when formulating strategies to improve inclusive maternal health care.

Women who were exposed to mass media (radio and newspapers) had higher odds of having childbirth at a health facility compared to those who were not exposed. This finding is consistent with studies conducted in Bangladesh, Guinea and Eritrea [42, 43, 49]. Mass media can promote healthy behavioral changes by frequently broadcasting programs and public service announcements that describe the benefits of health facility childbirths and other maternal healthcare services [50]. This enables positive changes in women’s attitudes, social norms and behavior that may lead to increased utilization of health facilities for childbirths [43]. Mass media also offer health information services such as announcements of locations and working hours of health facilities with free maternal healthcare services which encourage women and their partners to take practical action towards healthy behavioral changes [50, 51]. The role of mass media in women’s choice of place of delivery calls for enhancement in easier and cheaper access to media.

The study results also indicated that women who had no big problems regarding the distance to the nearby health facility had higher odds of having a health facility-based childbirth compared to women who had big problems accessing health facilities. Distance to health facilities has been found to be an important determinant of place of delivery in other similar contexts [1, 27, 49]. Since over 75% of the women in Uganda live in rural areas, proximity to a health facility is likely to affect use of the available services. Distance has a direct impact on the choice of the place of childbirth due to transportation challenges in terms of availability and affordability [49]. Hence women who are unable to afford these costs will end up having home childbirths. Thus, concerned stakeholders need to explore sustainable solutions to health facility access barriers such as maternity waiting homes and construction of more maternal health facilities.

### Strengths

Standardized procedures are a requirement of DHS surveys in data collection and validated questionnaires are used which ensures the internal and external validity of the results.

Secondly, we used the most recent nationally representative sample and weighed the data for analysis and therefore our results are generalizable to all Ugandan women aged 15 to 49 years.

### Limitations

Most data on the predictors were based on self-reports and could not be verified through records given this was cross sectional study. This carries a risk of social desirability bias. Additionally, the data could be affected by recall and interviewer biases. However, we do not anticipate these biases to affect the observed estimates because our study was based on secondary data collected through the robust validated DHS surveys. Data on explanatory variables such as wealth, residence reflected the women’s conditions at the time of the survey and not at the time of childbirth and hence women may have moved from one category of classification into another. Whereas the possibility of the study participants crossing from one category to another exists, it does not affect the overall interpretation and implications of our findings, especially as there is an equal chance of backward movement as well.

## Conclusion

Our study has revealed that health facility utilization during childbirth is higher relative to other countries in the East African region and it is associated with age, level of education, wealth index, residence, region, ANC utilisation, exposure to mass media, distance to health facilities, religion and ethnicity. The identified predictors act on both the supply and demand side for health care services, and thus emphasizes the significance of the social determinants of health as well as the need for programs/ interventions that focus beyond improving physical access. Rural areas should be targeted to address the barriers to health facility delivery. Implementation of proven interventions to improve utilization of health facility delivery should be tailored according to geographical regions and education level.

Given that Uganda has free health care services, the study showed that poorer women are less likely to utilize health facility delivery. Studies to evaluate different economic interventions targeting the poorer women are warranted. Taken together, it is recommended that in order to increase health facility deliveries in Uganda, maternal health programs should be promoted to target the poor, less educated and older women especially those residing in rural areas with limited exposure to mass media.

## Data Availability

Access to the DHS data sets is openly available upon requests made to MEASURE DHS on their website (URL: https://www.dhsprogram.com/data/available-datasets.cfm).

## Abbreviations

EA: Enumeration area
AOR: Adjusted Odds Ratio
CI: Confidence Interval
COR: Crude Odds Ratio
DHS: Demographic Health Survey
UDHS: Uganda Demographic Health Survey
OR: Odds Ratio
SD: Standard Deviation
WHO: World Health Organization
SPSS: Statistical Package for Social Science
USAID: United States Agency for International Development
